# Lipopolysaccharide and HMGB1: key regulatory factors in the pathophysiology of sepsis a mechanistic and therapeutic review

**DOI:** 10.3389/fimmu.2026.1862196

**Published:** 2026-06-26

**Authors:** ZiAng Wang, ZhengGang Luan

**Affiliations:** Department of Critical Care Medicine, The First Hospital of China Medical University, Shenyang, China

**Keywords:** caspase-11, high mobility group box 1, lipopolysaccharide, sepsis, toll-like receptor 4

## Abstract

Sepsis is a life-threatening clinical syndrome characterized by high morbidity and mortality. In the pathogenesis of Gram-negative bacterial infection, lipopolysaccharide (LPS) functions as a critical pro-inflammatory toxin that initiates inflammatory and coagulation cascades through two principal receptor systems: Toll-like receptor 4 (TLR4), expressed on the cell surface and within endosomes, and the cytosolic inflammatory caspases — caspase-11 in mice and caspases-4 and -5 in humans. In the extracellular environment, LPS binds to high mobility group box 1 protein (HMGB1) to form an HMGB1–LPS complex, which is internalized through receptor for advanced glycation end-products (RAGE)–mediated endocytosis and trafficked to the lysosome. Within the acidic lysosomal compartment, HMGB1 permeabilizes the limiting membrane, enabling LPS to access and activate caspase-11. This cascade drives further HMGB1 release, amplifies inflammation and coagulopathy, and ultimately contributes to multi-organ failure. The observation that LPS-driven fulminant inflammation depends critically on HMGB1 cooperation has opened new therapeutic avenues directed at HMGB1 and has yielded encouraging results in preclinical models. However, no such strategy has yet been translated into clinical practice. In addition, HMGB1 can be actively released by peripheral sensory neurons following tissue injury, a mechanism now recognized as integral to the initiation and propagation of inflammation. The present review synthesizes current understanding of the reciprocal interactions between LPS and HMGB1 and considers emerging therapeutic opportunities in sepsis.

## 1 Introduction

Sepsis is a severe systemic disorder triggered by bacterial infection, in which dysregulation of the host inflammatory response leads to dysfunction of multiple vital organ systems, most notably the respiratory, cardiovascular, renal, nervous, and coagulation systems (1, 2). Although timely antimicrobial therapy, fluid resuscitation, and respiratory support can improve survival, substantial improvement in clinical outcomes still requires the identification of novel and more effective therapeutic strategies.

LPS, abundantly expressed on the outer membrane of Gram-negative bacteria, is one of the most potent pro-inflammatory molecules known and constitutes a core pathogenic factor in sepsis (3). During severe Gram-negative infection, two distinct LPS-sensing systems operate. The first relies on the cell-surface and endosomal receptor complex comprising TLR4 and myeloid differentiation factor 2 (MD-2), which is widely distributed across cell types and plays a pivotal role in initiating innate immune responses (4). The second is a cytosolic sensing system mediated by caspase-11 in mice and caspases-4 and -5 in humans (5, 6). Activation of these inflammatory caspases triggers a cascade that includes pro-inflammatory cytokine release, cleavage of Gasdermin D(GSDMD), induction of pyroptosis, and disruption of coagulation homeostasis (7). This process is particularly prominent in myeloid cells, endothelial cells, and selected additional cell populations. Engagement of extracellular LPS with MD-2 within the TLR4 complex requires the accessory proteins CD14 and LPS-binding protein, while access of LPS to the cytosolic sensor caspase-11 further depends on HMGB1,a protein that was early recognized as both a pro-inflammatory cytokine and a therapeutic target (8, 9) and subsequently recognized as the prototypical damage-associated molecular pattern (DAMP) (10). Notably, activation of either TLR4 or caspase-11 promotes extracellular release of HMGB1 (11) (12).

TLR4-deficient (Tlr4-/-) mice exposed to lethal doses of LPS together with agents that promote endogenous type I interferon production are not protected; in contrast, caspase-11-deficient (Casp11-/-) mice display marked resistance to lethal septic shock (in murine LPS challenge models) (5, 6). Type I interferon is a particularly effective inducer of HMGB1 release, and the released HMGB1 forms heteromeric complexes with LPS that undergo RAGE-mediated endocytosis, ultimately leading to caspase-11 activation(murine caspase-11; human orthologs: caspase-4 and caspase-5) (11). Collectively, these murine findings suggest that cytosolic LPS sensing via caspase-11 may represent an important and previously underappreciated driver of severe sepsis and septic shock. However, these data derive principally from murine endotoxemia models; the relative contributions of TLR4 and cytosolic caspase-4/5 pathways in human clinical sepsis remain to be determined, and both systems likely cooperate in a context-dependent manner.

On the basis of current evidence, this review defines the LPS-HMGB1-caspase-11/4/5 axis as an important mechanism of inflammatory amplification in sepsis, rather than as a simple replacement for canonical TLR4 signalling. This pathway can be summarized as follows: extracellular LPS forms complexes with HMGB1; these complexes undergo RAGE-mediated endocytosis and traffic to endolysosomal compartments; HMGB1 promotes lysosomal membrane permeabilization under acidic conditions; cytosolic LPS activates caspase-11 in mice or caspases-4/5 in humans; and downstream GSDMD cleavage drives pyroptosis, IL-1β/IL-18 release, coagulation disturbances, and organ injury. Importantly, many mechanistic findings supporting this pathway derive from murine endotoxemia models. Their interpretation in human sepsis should therefore be constrained by pathogen type, infection site, immune status, metabolic context, and disease stage.

**Figure 1:**
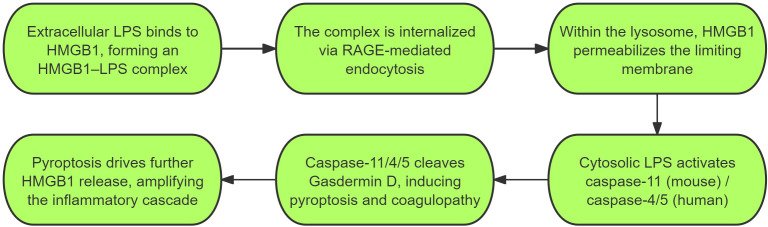
Schematic overview of the feed-forward HMGB1–LPS–caspase-11/4/5–Gasdermin D axis: extracellular LPS is captured by HMGB1, internalized via RAGE, liberated from lysosomes, and then sensed by cytosolic caspase-11/4/5, which cleaves Gasdermin D to drive pyroptosis and coagulopathy, further promoting HMGB1 release and amplifying the inflammatory cascade.

To guide the reader, the core mechanistic axis reviewed here can be summarized as follows: (1) Extracellular LPS binds to HMGB1, forming an HMGB1–LPS complex; (2) The complex is internalized via RAGE-mediated endocytosis; (3) Within the lysosome, HMGB1 permeabilizes the limiting membrane; (4) Cytosolic LPS activates caspase-11 (mouse)/caspase-4/5 (human); (5) Caspase-11/4/5 cleaves Gasdermin D, inducing pyroptosis and coagulopathy; (6) Pyroptosis drives further HMGB1 release, amplifying the inflammatory cascade (**Figure 1)**. The following sections address each node of this pathway in detail.

## 2 Extracellular release of HMGB1

**Figure 2:**
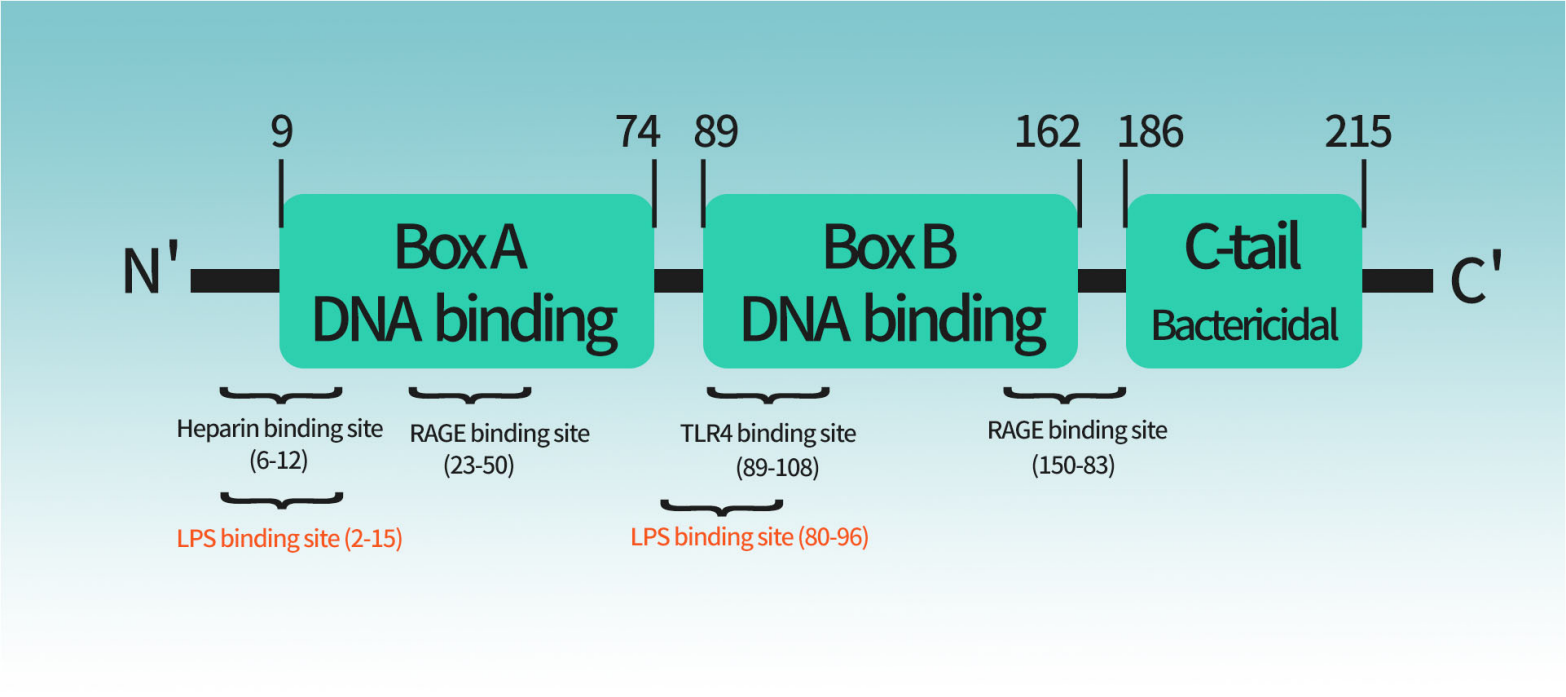
The structure of HMGB1 and the locations of its binding sites for TLR4, RAGE, heparin, and LPS. The HMGB1 structure depicted is derived from UniProt entry P09429 (human HMGB1). Visualization was generated using BioRender based on the AlphaFold2 predicted structure (AlphaFold DB: AF-P09429-F1).

HMGB1 is a nuclear protein ubiquitously expressed across animal cells. Its coding sequence comprises 215 amino acid residues arranged to form two positively charged DNA-binding domains and a negatively charged C-terminal tail (**Figure 2**) (8, 13). Within the nucleus, HMGB1 participates in transcriptional regulation, nucleosome assembly, DNA repair, and the maintenance of chromatin architecture. Global genetic ablation of HMGB1 is embryonically lethal, and the protein is highly conserved in evolution — sequence identity among mammals approaches 99% — underscoring its fundamental biological importance. To ensure nuclear localization under homeostatic conditions, HMGB1 contains two nuclear localization signals (NLSs) that are indispensable for its subcellular distribution and function.

Once released into the extracellular space, HMGB1 exerts two mechanistically and functionally distinct roles. First, in its disulfide isoform (Cys23-Cys45 disulfide bond, Cys106 reduced), HMGB1 functions as a direct pro-inflammatory cytokine: it binds MD-2 within the TLR4 receptor complex with high affinity, triggering NF-κB activation and downstream cytokine release independently of any bound partner ligand. Second, and of central focus in this review, HMGB1 — regardless of redox state — functions as a molecular carrier that binds LPS in the extracellular milieu and facilitates its delivery to cytosolic caspase-11 via RAGE-mediated endocytosis and lysosomal escape. This second function, which is mechanistically distinct from canonical TLR4 signalling and requires HMGB1 cooperation, represents the primary novel axis discussed herein.

Active extracellular release of HMGB1 in response to LPS and other stimuli requires post-translational modification of the nuclear protein to permit its translocation into the cytoplasm and subsequent secretion. A key modification is hyperacetylation of specific lysine residues within the NLSs, which disrupts the constitutive nucleocytoplasmic shuttling of HMGB1 and promotes its cytoplasmic accumulation, thereby priming it for extracellular release (14). Nuclear hyperacetylation is achieved through the combined upregulation of histone acetyltransferase (HAT) activity and suppression of histone deacetylase (HDAC) activity (15–17).

**Figure 3:**
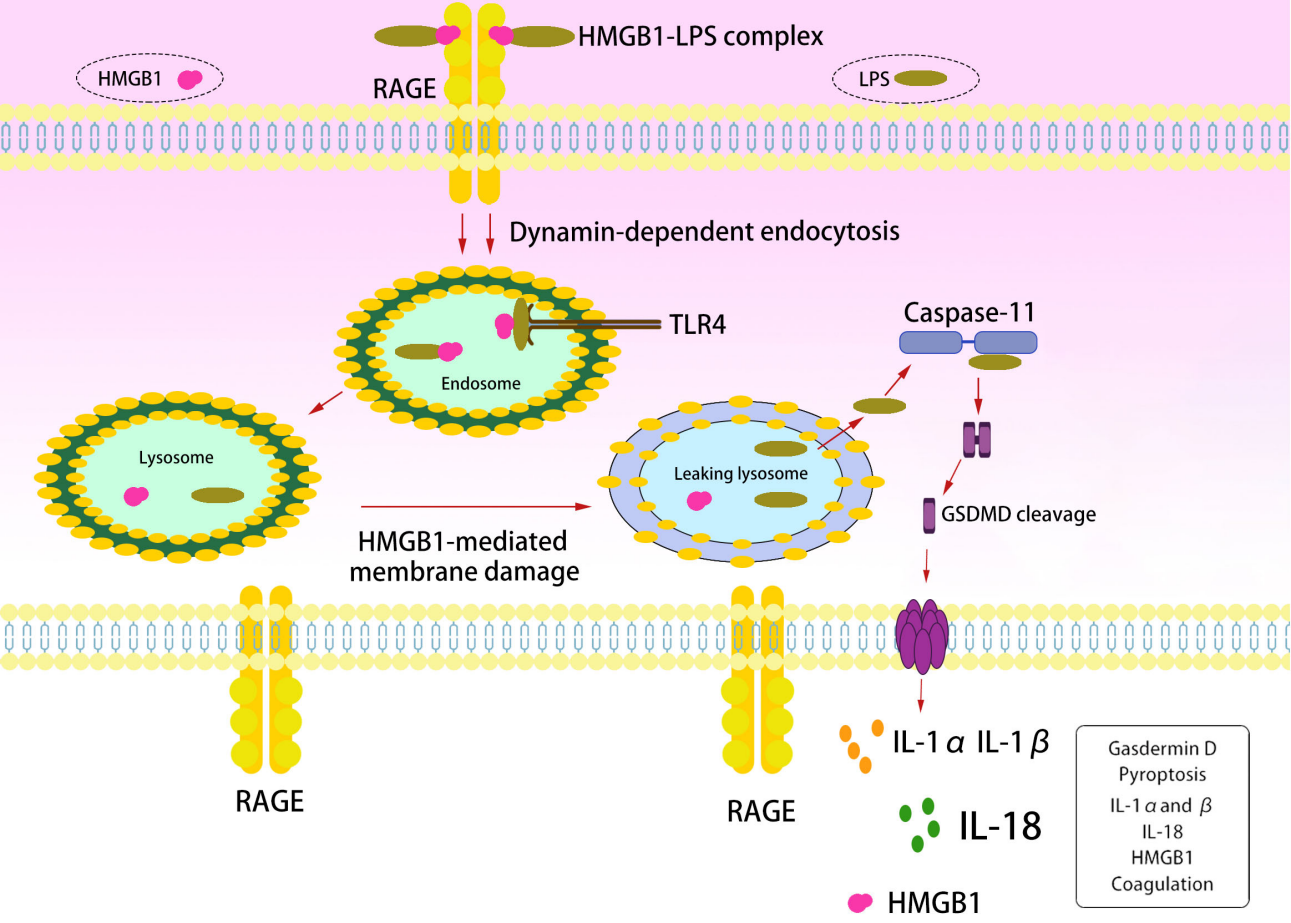
HMGB1 binds to LPS in the extracellular milieu to form an HMGB1-LPS complex, which is subsequently internalized via RAGE-mediated endocytosis and delivered into the cytoplasm, where it activates the intracellular LPS sensor caspase-11, thereby triggering inflammatory and coagulatory responses.

During endotoxaemia, systemic accumulation of HMGB1 occurs substantially later than that of most early inflammatory mediators induced by LPS. Specifically, serum HMGB1 becomes detectable no earlier than 8 hours after LPS exposure and subsequently rises to a prolonged plateau over 16–32 hours (8). Cytosolic HMGB1 is released extracellularly through multiple mechanisms, and the released protein exerts its biological effects via a range of receptors, including RAGE (18). Myeloid and endothelial cells internalize extracellular LPS–HMGB1 complexes through RAGE, engaging two LPS-specific binding sites (19). These complexes are delivered to lysosomes, where the acidic environment enables HMGB1 to permeabilize the lysosomal membrane, releasing LPS into the cytoplasm, where it engages caspase-11 (20). This interaction drives GSDMD cleavage and oligomerization, leading to the formation of plasma-membrane nanopores and the induction of pyroptosis. Activities within both live and dying cells facilitate the release of interleukins such as IL-1α, IL-1β, and IL-18, together with HMGB1 (**Figure 3)** (21, 22). Importantly, the observation that caspase-1/caspase-11 double-deficient mice exhibit markedly reduced systemic HMGB1 levels establishes pyroptosis as a principal route of HMGB1 release during sepsis (23). Myeloid cells additionally secrete bioactive HMGB1 via exocytosis of secretory lysosomes, a pathway that is also involved in IL-1β secretion, although HMGB1 and IL-1β are stored in distinct vesicle populations (24).

Notably, the principal cellular source of circulating HMGB1 in sepsis is neither macrophages nor endothelial cells but hepatocytes (12). Hepatocyte-specific deletion of HMGB1 substantially improves survival in lethal endotoxaemia and prevents the accumulation of circulating HMGB1, whereas myeloid-specific deletion neither lowers systemic HMGB1 nor confers survival benefit (11). Hepatocytes and inflammatory cells thus release HMGB1 via distinct receptor-dependent pathways (12): hepatocytes use surface-expressed TLR4 to recognize and internalize extracellular LPS and deliver it to caspase-11, which in turn drives GSDMD cleavage and activation. These events do not cause hepatocyte pyroptosis but instead promote calcium-dependent phosphorylation of nuclear HMGB1, resulting in cytoplasmic accumulation and extracellular release via exosomes. LPS-dependent HMGB1 release from hepatocytes therefore requires coordinated engagement of both canonical LPS-sensing systems — TLR4 and caspase-11.

The receptor preference and downstream biological activity of HMGB1 depend on the redox state of three cysteine residues (Cys23, Cys45, and Cys106). In quiescent cells these cysteines remain fully reduced (13). However, the redox state of HMGB1 is dynamic and shifts according to the intracellular and extracellular redox balance, thereby modulating its immunological functions. Necrosis releases the fully reduced form, which forms heteromeric complexes with the chemokine CXCL12 (SDF-1) to exert chemotactic activity; this complex binds the CXCL12 receptor CXCR4 and initiates chemotaxis synergistically, beyond that achieved by CXCL12 alone (25). Mild oxidation produces a disulfide bond between Cys23 and Cys45, while Cys106 remains reduced. This modification converts extracellular HMGB1 into a potent pro-inflammatory cytokine that binds MD-2 within the TLR4 receptor complex with high affinity (26). Regardless of whether CXCL12 is present, the fully reduced form of HMGB1 cannot activate TLR4 signalling, whereas the disulfide form is unable to activate the CXCR4 pathway. Further oxidation of HMGB1 irreversibly alters its molecular structure by generating sulfonyl groups on any or all of the three cysteine residues, producing an isoform devoid of pro-inflammatory activity. Recent findings in tumour biology indicate that sulfonyl HMGB1 functions as an anti-inflammatory molecule that, via RAGE signalling, recruits immunosuppressive cells-including regulatory T cells, M2-polarized macrophages, and myeloid-derived suppressor cells (27). Sulfonyl HMGB1 can also downregulate the function of antigen-presenting cells, including both conventional and plasmacytoid dendritic cells. Whether sulfonyl HMGB1 contributes to the immunosuppressive phase that emerges during and after sepsis remains to be formally demonstrated. We suggest that this question can be addressed through three complementary approaches. First, redox−preserving mass spectrometry, such as iodoacetamide alkylation followed by LC−MS/MS, could be applied to systematically quantify all three HMGB1 redox isoforms in serial plasma samples collected from sepsis patients across the full disease trajectory. Second, sulfonyl HMGB1 abundance could be correlated with established biomarkers of immunosuppression, including monocyte HLA−DR expression and lymphocyte apoptosis indices. Third, the functional properties of recombinant sulfonyl HMGB1 could be tested in ex vivo human immune cell assays. If sulfonyl HMGB1 indeed accumulates during the late, immunosuppressive phase of sepsis, this accumulation would represent a previously unrecognized mechanism that contributes to the heightened susceptibility to secondary infections, a hallmark of late sepsis mortality.

Although at least fourteen receptor systems have been proposed to recognize HMGB1, only TLR4 and RAGE play dominant roles in HMGB1-mediated inflammation. Other receptors are likely more relevant to complexes formed between HMGB1 and its partner ligands. As noted, HMGB1 binds not only LPS but also DNA, RNA, histones, nucleosomes, IL-1α, and IL-1β, among other immunostimulatory molecules — each of which is a high-affinity ligand for distinct HMGB1-associated receptors (28). HMGB1 heteromeric complexes enter the endosome through RAGE-mediated endocytosis, where certain HMGB1-bound molecules stimulate various TLRs. The complexes are then trafficked to lysosomes; under acidic conditions the complexes dissociate, HMGB1 disrupts the lysosomal membrane, and the liberated damage-associated and pathogen-associated molecular patterns (DAMPs and PAMPs) gain access to cytosolic pro-inflammatory sensors, including the inflammasomes, cGAS, and RIG-I.

Additional release mechanisms include platelet-derived HMGB1 following vascular injury (29, 30), lactate-induced macrophage HMGB1 acetylation and exosomal release in polymicrobial sepsis (31), and age-dependent decline of SIRT1 activity, which is thought to account for heightened inflammatory responses in elderly patients (32). The precise mechanisms underlying lactate-stimulated HMGB1 release require further investigation.

## 3 Role of HMGB1 in sepsis-induced organ failure

Extensive preclinical investigation of sepsis and endotoxaemia provides compelling evidence that HMGB1 and LPS, through a series of complex and interwoven mechanisms, jointly drive tissue injury and consequent multi-organ failure. These mechanisms span several biological levels, including amplified inflammation, aberrant immune cell activation, increased apoptosis, vascular endothelial injury, and dysregulated cellular energy metabolism, and they constitute an indispensable component of sepsis pathophysiology. A detailed understanding of the specific roles and interrelationships of HMGB1 and LPS in these processes is therefore essential for the development of effective therapeutic strategies aimed at mitigating tissue injury and preserving organ function (33).

### 3.1 HMGB1 and acute lung injury

A prominent feature shared by HMGB1-induced acute pathologies is its role as a key mediator of epithelial and endothelial barrier disruption. Specifically, HMGB1 binds RAGE and activates the Rho-kinase 1 signalling pathway, a process particularly consequential in the lung, where RAGE is constitutively expressed at high levels, unlike most other tissues (34). This interaction disrupts the pulmonary endothelial barrier and increases its permeability, representing an important mechanism in pathological states such as acute respiratory distress syndrome (ARDS). HMGB1 concentrations in bronchoalveolar lavage fluid from ARDS patients are markedly higher than corresponding plasma levels, further supporting its central role in lung injury. Sepsis-induced acute lung injury compromises gas exchange and is accompanied by intense inflammation, increased protein permeability, and severe damage to alveolar and endothelial cells, culminating in pulmonary dysfunction (35). Experimental work demonstrates that intratracheal administration of HMGB1 exacerbates these pathological changes, including neutrophil infiltration, interstitial oedema, and pro-inflammatory cytokine release, whereas anti-HMGB1 antibodies effectively ameliorate endotoxin-induced ARDS. The therapeutic effect is particularly pronounced for late-phase inflammation and occurs without altering the release of early mediators such as TNF-α and IL-1, consistent with the status of HMGB1 as a late-phase mediator (36).

In addition, HMGB1 further aggravates sepsis-related respiratory dysfunction by impairing neutrophil bactericidal capacity and inhibiting NADPH oxidase activity. Anti-HMGB1 antibody treatment significantly restores neutrophil function and mitigates sepsis-induced impairment of neutrophil NADPH oxidase activity. In acute lung injury, HMGB1 also reorganizes the actin cytoskeleton of endothelial cells, promoting a contractile phenotype that disrupts the endothelial barrier — a hallmark of pulmonary injury (37). Of note, HMGB1 excess can also induce pyroptosis of pulmonary macrophages, a programmed form of cell death accompanied by intense inflammation and coagulation activation that further exacerbates lung injury and dysfunction.

### 3.2 HMGB1 and acute myocardial injury

Sepsis impairs left ventricular function, reduces cardiac output and blood pressure, and elevates heart and respiratory rates. LPS-induced elevations in HMGB1 compromise systemic circulation primarily by disrupting myocardial contractile performance and impairing vascular endothelial integrity. Through TLR4-mediated signalling, LPS induces cardiomyocytes to increase HMGB1 expression and secretion *in vivo*. In murine endotoxaemia models, LPS-induced depression of myocardial contractility can be effectively prevented by a range of HMGB1 antagonists (38). HMGB1 disrupts tight junctions, reorganizes the actin cytoskeleton, and triggers the release of large quantities of cytokines and chemokines, thereby increasing microvascular permeability; it also promotes expression of surface adhesion molecules such as intercellular adhesion molecule 1 (ICAM-1) and vascular cell adhesion molecule 1 (VCAM-1), further amplifying these effects (34). In several experimental sepsis studies, HMGB1-specific antagonist therapy has successfully reversed endothelial barrier damage.

### 3.3 HMGB1 and acute kidney injury

In sepsis, endotoxaemia is a well-established complication that can precipitate acute kidney injury (39). Experimental evidence indicates that LPS directly injures podocytes — a critical component of the glomerular filtration barrier — thereby impairing renal function, and that reducing HMGB1 expression attenuates endotoxin-induced podocyte injury. Renal tubular epithelial cells exposed to HMGB1 additionally develop mitochondrial dysfunction (40). During sepsis, HMGB1 accumulates within renal tissue and is excreted into the urine, a process that facilitates HMGB1–TLR4 interactions and converts tubular epithelial cells into pro-inflammatory effectors. SIRT1-mediated deacetylation of HMGB1 has been shown to suppress HMGB1 release and to ameliorate sepsis-associated acute kidney injury (41). This observation reinforces the central role of HMGB1 in the pathophysiology of acute kidney injury and highlights the therapeutic potential of interventions directed at HMGB1 and its downstream signalling pathways.

### 3.4 HMGB1 and coagulopathy

Coagulopathy is a hallmark of sepsis, ranging from subclinical hypercoagulability with a predisposition to venous thromboembolism to overt and life-threatening disseminated intravascular coagulation (DIC). The pathophysiology of DIC involves extensive microvascular thrombosis accompanied by consumption of platelets and coagulation factors. Coagulation is an integral arm of innate immunity, originally evolved to trap and confine microorganisms within the microvasculature; however, once the microvasculature is compromised, localized infection may progress to sepsis. The initiating step involves LPS, with the assistance of HMGB1, gaining access to the cytoplasm and activating caspase-11, which in turn catalyses GSDMD cleavage and the formation of membrane-perforating peptides that generate nanopores in the plasma membrane. In the context of coagulation, these pores in macrophages promote calcium influx rather than pyroptosis (42, 43). The resulting surface changes enhance tissue factor activity and facilitate assembly of cofactor–protease complexes on vascular endothelial cells, thereby initiating the extrinsic coagulation cascade. Extracellular HMGB1 further upregulates tissue factor expression in endothelial cells via TLR4 signalling and activation of transcription factors such as NF-κB and early growth response protein 1 (Egr-1) (44). An additional mechanism underlying LPS- and HMGB1-induced coagulopathy involves platelet biology: HMGB1 is normally stored within platelet granules, and upon platelet activation it is translocated to the cell surface, where it potentiates platelet activation in a TLR4-dependent manner, manifested by increased surface HMGB1 expression and enhanced platelet aggregation (45). Platelet-released HMGB1 has been identified as a critical promoter of thrombosis, and experimental data indicate that mice with platelet-specific HMGB1 deficiency exhibit reduced thrombus formation, platelet aggregation, inflammation, and organ injury (46). Platelet-derived HMGB1 therefore hypothesized to play a significant role in the microvascular thrombosis characteristic of sepsis.

### 3.5 HMGB1 and neurological injury

Rising systemic HMGB1 concentrations disrupt the blood–brain barrier, permitting pro-inflammatory molecules such as LPS and HMGB1 to enter the central nervous system and initiate neuroinflammation. The resulting inflammatory response is both complex and multifaceted, producing a range of central nervous system dysfunctions, most notably significant cognitive decline (47). In murine models of sepsis-associated encephalopathy, systemic infection not only elevates extracellular levels of disulfide HMGB1 within the central nervous system but also activates NLRP3 via the TLR4–MD-2 complex, thereby aggravating neuroinflammation and cognitive impairment. These findings underscore the central role of HMGB1 in mediating cognitive dysfunction in septic patients and suggest that anti-HMGB1 antibody therapy has been shown to contribute to represent an effective strategy to prevent or reverse sepsis-related cognitive decline (48).

Post-surgical cognitive decline, including memory impairment, is a common complication — particularly in patients over 65 years of age and in aged experimental animals (49). Experimental evidence indicates that HMGB1-specific antagonists can effectively prevent postoperative neuroinflammation and cognitive decline (50), further highlighting the pivotal role of HMGB1 in neuroinflammation arising from both sterile and infection-driven systemic inflammation.

**Figure 4:**
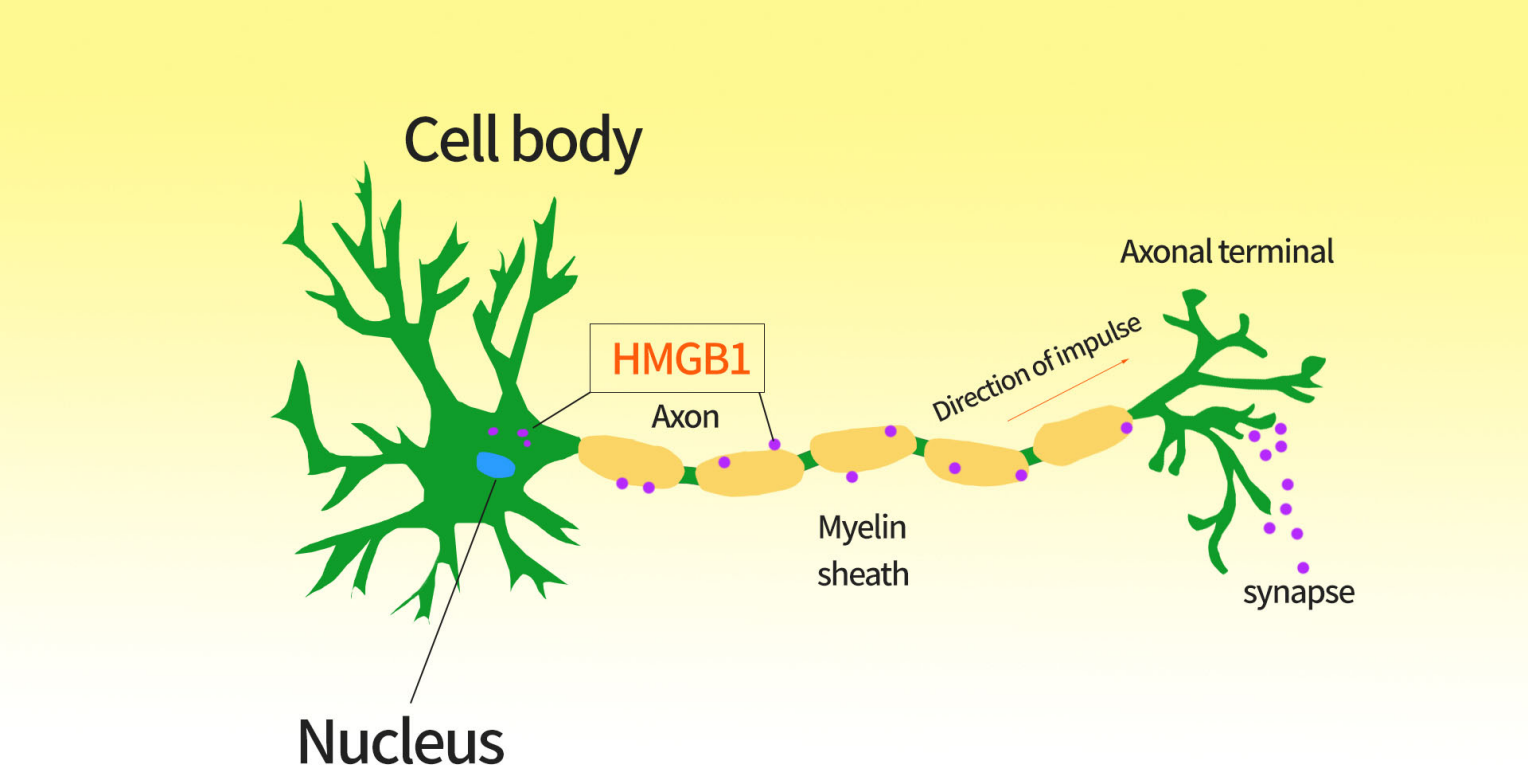
Activation of nociceptive neurons triggers the retrograde release of HMGB1 within their innervated regions.

Recent advances have also revealed a novel role for sensory neurons in inflammation: these cells can actively release HMGB1 and thereby elicit peripheral inflammation. Activation of nociceptive neurons induces retrograde release of HMGB1 within their innervation territory, triggering local inflammation (**Figure 4)** (51). This finding underscores the importance of neurogenic HMGB1 in the development of neuroinflammation. Experimental studies show that neuron-specific deletion of HMGB1 or administration of neutralizing anti-HMGB1 antibodies markedly ameliorates inflammation and tissue injury in experimental polyarthritis and nerve injury models (48). Nociceptor-derived HMGB1 is therefore considered a key regulator of neuroinflammatory responses to diverse forms of tissue injury, although its specific role in sepsis requires further investigation.

In the context of sepsis, peripheral neuronal HMGB1 release is proposed to contribute to sepsis−associated encephalopathy through at least two complementary mechanisms. The first is direct hematogenous spread, whereby neuronal HMGB1 released into the systemic circulation crosses the disrupted blood–brain barrier, and subsequently activates microglial TLR4/RAGE signalling to amplify central neuroinflammation. The second is neuroimmune reflex modulation, where sensory input via the vagus nerve influences macrophage activation through the cholinergic anti−inflammatory pathway, and neuronal HMGB1 may modulate this circuit. Should the existing evidence be insufficient to establish a definitive causal link to sepsis, we explicitly acknowledge this limitation and present neuronal HMGB1 release as a promising yet preliminary avenue that warrants dedicated investigation in sepsis models.

### 3.6 Common mechanistic themes in HMGB1-mediated organ injury

Despite the apparent organ-specificity of the injuries described above, a review of the evidence reveals four recurring mechanistic themes that underlie HMGB1-mediated damage across multiple organ systems. The first is endothelial and epithelial barrier disruption via RAGE-ROCK1 signalling, a pathway that is most prominent in the lung, a site of constitutively high RAGE expression, but also operates in cardiac microvascular injury and blood-brain barrier disruption. The second is pyroptosis-driven inflammation mediated by caspase-11 and GSDMD; this mechanism is active in macrophages within pulmonary, renal, and hepatic compartments, where the resulting GSDMD nanopores drive both cytokine release and secondary HMGB1 release, thereby creating a positive feedback loop. The third involves coagulopathy and microthrombosis, as HMGB1 promotes coagulation through tissue factor upregulation via the TLR4-NF-κB pathway and through platelet HMGB1-mediated aggregation, affecting multiple vascular beds simultaneously. The fourth theme is mitochondrial dysfunction, which has been observed in renal tubular epithelial cells and cardiomyocytes; however, the precise HMGB1-dependent mechanism, whether it acts through RAGE, TLR4, or intracellular pathways, remains incompletely defined and therefore represents a priority for future investigation.

## 4 Emerging therapeutic strategies for sepsis

### 4.1 Unfractionated heparin therapy

The observation that LPS requires HMGB1 to exert its full toxic effect in sepsis has provided an important foundation for novel therapeutic strategies, principally aimed at blocking LPS uptake into cells and its interaction with caspase-11, thereby attenuating downstream inflammation (52).

Heparin, a mammalian polysaccharide, has been shown to bind specifically to defined sites on HMGB1, effectively preventing LPS–HMGB1 association and, consequently, the formation of LPS–HMGB1 complexes (53). This binding not only diminishes LPS toxicity but also inhibits caspase-11 activation, thereby reducing endotoxin-induced inflammation and coagulopathy. In addition, heparin binds extracellular histones and mitigates histone-mediated cytotoxicity, another important pathogenic pathway in severe sepsis. Furthermore, heparin has been shown to counteract HMGB1-mediated disruption of endothelial barrier integrity by preventing the downregulation of vascular endothelial cadherin, a key component of endothelial adherens junctions (54). These findings highlight the pleiotropic potential of heparin in sepsis therapy, particularly through non-anticoagulant mechanisms that attenuate inflammation and cellular injury. Importantly, the doses required to achieve these therapeutic effects are substantially lower than those needed for anticoagulation, offering the potential to reduce bleeding risk (52–54). To optimize the design of future clinical sepsis trials, both the dose and the type of heparin used merit careful consideration. Low-dose heparin or non-anticoagulant modified heparin may represent an effective strategy, preserving anti-inflammatory and cytoprotective activity while minimizing the risk of haemorrhagic complications.

In summary, therapeutic strategies targeting the LPS–HMGB1 axis, such as low-dose or non-anticoagulant heparin, offer a promising new direction for sepsis management. Future research should further investigate the clinical translation of these approaches and should consider patient stratification based on pathogen type to optimize efficacy and minimize adverse effects.

### 4.2 HMGB1-neutralizing antibody therapy

During sepsis, endogenous autoantibodies against HMGB1 are generated, and their presence is associated with favourable clinical outcomes in patients with septic shock (55). Moreover, polyclonal and monoclonal anti-HMGB1 antibodies have demonstrated significant efficacy in numerous preclinical studies of sepsis. A challenge for antibody-based therapy directed at HMGB1-mediated pathology, however, is that HMGB1 may form complexes with partner molecules (such as DAMPs and PAMPs), and the resulting steric hindrance can interfere with antibody recognition and thereby reduce therapeutic efficacy. Notably, the relatively late kinetics of HMGB1 release during sepsis provide a unique therapeutic window in which antagonistic antibody therapy remains feasible. Specifically, HMGB1 release peaks 16–32 hours after LPS exposure, and administration of anti-HMGB1 monoclonal antibodies within 24 hours of sepsis onset in the caecal ligation and puncture (CLP) model yields satisfactory outcomes. In contrast, treatment delivered later in the same model — for example, 36 hours after CLP — is compromised by the accumulation of extracellular PAMPs and DAMPs, including DNA, RNA, histones, and nucleosomes, which bind extracellular HMGB1 tightly and thereby attenuate antibody efficacy (56).

The timing of HMGB1-directed antibody therapy is therefore critical. Early identification of patients and administration of antibodies capable of recognizing HMGB1 epitopes not yet occupied by LPS or other partner ligands could afford optimal therapeutic opportunity, consistent with the favourable outcomes observed in preclinical sepsis studies. This indicates that, through precise modulation of treatment timing and antibody specificity, HMGB1-directed therapy holds broad promise for the treatment of sepsis and related inflammatory diseases.

### 4.3 Prevention of HMGB1-mediated lysosomal disruption

A phenotypic screening study of diverse synthetic compounds aimed at inhibiting the LPS–HMGB1–caspase-11 signalling axis successfully identified a porphyrin-class small molecule, FeTPPS. This compound demonstrates the ability to block caspase-11 activation and thereby suppress the downstream inflammatory and coagulation cascades (57). Of particular note, FeTPPS exhibits substantial potential to prevent lethality in experimental sepsis. Mechanistically, it operates by disrupting LPS–HMGB1 binding and by attenuating the capacity of HMGB1 to permeabilize the lysosomal membrane.

Through these dual and complementary mechanisms, FeTPPS effectively reduces aberrant cytosolic delivery of LPS within macrophages, offering a novel strategy and renewed hope for the treatment of related diseases.

Taken together, the three therapeutic approaches discussed above, namely low-dose heparin, anti-HMGB1 neutralizing antibodies, and FeTPPS, each target distinct nodes of the LPS–HMGB1–caspase-11 axis but differ substantially in their mechanism, developmental stage, and limitations (**Table 1)**. Heparin offers the advantage of pleiotropic activity and immediate clinical availability but carries bleeding risk even at low doses, and its optimal dosing strategy for this specific indication remains undefined. Anti-HMGB1 antibodies provide high specificity but are constrained by a narrow therapeutic window (approximately 24 hours post-onset) and reduced efficacy in the presence of accumulated extracellular DAMPs and PAMPs that compete for HMGB1 epitopes. FeTPPS, while mechanistically elegant in its dual mechanism of action, remains in early preclinical development with no pharmacokinetic or safety data in humans. Given these complementary profiles, combination strategies, such as heparin to block LPS–HMGB1 complex formation combined with an anti-HMGB1 antibody to neutralize free extracellular HMGB1, warrant investigation in future preclinical studies.

**Table 1:** Summary of emerging therapeutic strategies targeting the LPS–HMGB1 axis in sepsis.

Strategy	Molecular target	Mechanism of action	Stage of development	Advantages	Limitations
Unfractionated Heparin (low-dose)	HMGB1 heparin-binding sites; extracellular histones (52, 53)	Blocks LPS-HMGB1 complex formation; inhibits caspase-11 activation; neutralizes histone toxicity (54)	Preclinical (mouse endotoxemia); retrospective clinical associations	Pleiotropic; already in clinical use; low cost	Bleeding risk even at low dose; optimal dose/type undefined; no RCT evidence for this specific mechanism
Anti-HMGB1 Neutralizing Antibodies	Extracellular HMGB1	Prevents HMGB1-RAGE/TLR4 interaction; blocks LPS-HMGB1 endocytosis (55, 56)	Preclinical (CLP and endotoxemia models); no human trials	Narrow therapeutic window (late mediator); high specificity	Steric hindrance by DAMPs/PAMPs; 24-h window limits applicability; manufacturing cost; no clinical data (56)
FeTPPS (porphyrin small molecule)	HMGB1-LPS interaction; lysosomal membrane	Disrupts LPS-HMGB1 binding; prevents HMGB1-mediated lysosomal permeabilization; blocks caspase-11 (57)	Preclinical only	Dual mechanism; novel mechanistic approach	Off-target effects unknown; no pharmacokinetic/safety data in humans; early discovery stage (57)

## 5 Discussion

The central message of this review is that HMGB1 is not only a late inflammatory mediator in sepsis but also a key bridge linking extracellular LPS to cytosolic inflammatory caspase activation. By forming complexes with LPS and promoting RAGE-mediated endocytosis, HMGB1 enables LPS to enter lysosomal compartments and subsequently escape into the cytosol, where it activates the caspase-11/4/5-GSDMD axis. This mechanism provides a framework for understanding LPS-induced pyroptosis, cytokine release, coagulation disturbances, and organ injury.

Current literature supports an important role for HMGB1 in endotoxemia and experimental sepsis, but the translational boundaries among different models must be clearly defined. Murine lethal LPS models are useful for revealing extreme caspase-11-dependent inflammatory amplification, whereas caecal ligation and puncture models better approximate polymicrobial infection but still do not fully recapitulate immune paralysis, metabolic dysfunction, secondary infection, and therapeutic interventions in human sepsis. Human caspases-4/5 are functionally analogous to murine caspase-11, but their regulation and clinical relevance have not been validated to the same extent. Accordingly, cytosolic LPS sensing should be understood as an important complement to canonical TLR4 signalling rather than as a replacement for the TLR4-centered model.

The translational gap between rodent endotoxemia models and clinical sepsis remains a central challenge for the field. Human sepsis involves polymicrobial infections, variable host genetics, comorbidities, and a biphasic immune trajectory (hyperinflammation followed by immunosuppression) that no single animal model fully replicates.

### 5.1 Outstanding questions and future directions

Several key questions remain unanswered and should guide the next phase of research in this field.

#### 5.1.1 HMGB1 isoform specificity

The three redox isoforms of HMGB1 (fully reduced, disulfide, sulfonyl) exhibit distinct receptor specificities and biological activities. The relative abundance of these isoforms in human sepsis plasma has not been systematically quantified using redox-preserving mass spectrometry. Whether the immunosuppressive sulfonyl isoform accumulates during the late immunosuppressive phase of sepsis, and whether this accumulation worsens secondary infection susceptibility, remains unknown.

#### 5.1.2 Cell type-specific targeting

Hepatocyte-derived HMGB1 accounts for the majority of circulating HMGB1 in murine endotoxemia, yet this has not been validated in human sepsis. Developing cell type-specific HMGB1 inhibitors (e.g., liver-targeted nanoparticles) could improve therapeutic selectivity.

#### 5.1.3 Caspase-4/5 validation in human sepsis

Most mechanistic data on cytosolic LPS sensing derive from murine caspase-11 studies. The contribution of human caspase-4/5 to clinical sepsis pathology is undercharacterised; future studies using primary human macrophages, monocyte-derived cells, and clinical biomarkers of pyroptosis (e.g., circulating GSDMD-NT) are needed.

#### 5.1.4 Reasons for limited clinical success

Prior HMGB1-targeting strategies have not entered clinical use, owing to the molecular complexity of HMGB1, its multiple post-translational modifications, and the broad DAMP/PAMP competition for epitope access. Patient stratification by pathogen type (Gram-negative vs. polymicrobial), sepsis phase (hyperinflammatory vs. immunosuppressive), and HMGB1 isoform profile is essential to identify responders for future trials.

Future research should move beyond single animal models toward cross-model and cross-species validation. Specifically, primary human cells and clinical samples from patients with sepsis should be used to evaluate the relationships among LPS-HMGB1 complexes, HMGB1 redox status, RAGE-dependent endocytosis, caspase-4/5 activation, GSDMD cleavage, and clinical outcomes. Therapeutic studies should prioritize patient-stratification strategies, such as enrichment for Gram-negative infection, high circulating HMGB1, specific HMGB1 redox isoforms, or prominent coagulation abnormalities. Only by integrating mechanistic validation, patient selection, and safety assessment can therapies targeting the LPS-HMGB1 axis move from experimental models toward clinical application.
